# Perspectives on the care process by nurses working in Child and Adolescent Psychosocial Care Centers

**DOI:** 10.1590/0034-7167-2025-0156

**Published:** 2026-07-06

**Authors:** Fernanda da Costa Ferreira, Mariana Miguel Vieira, Ângela Maria Rosas Cardoso

**Affiliations:** I Fundação de Ensino e Pesquisa em Ciências de Saúde. Brasília, Distrito Federal, Brazil

**Keywords:** Mental Health, Nursing, Psychiatric Nursing, Biopsychosocial, Mental Health Services., Salud Mental, Enfermería, Enfermería Psiquiátrica, Modelos Biopsicosociales, Atención a la Salud Mental.

## Abstract

**Objectives:**

to understand nurses’ perception from the Federal District Health Department and Multidisciplinary Program in Child and Adolescent Mental Health residents regarding practices at the Child and Adolescent Psychosocial Care Center, identifying challenges and gaps in their training.

**Methods:**

semi-structured interviews were conducted and analyzed using thematic analysis, which consists of three phases: pre-analysis, content exploration, and elaboration of results.

**Results:**

55.5% participants have worked at the Child and Adolescent Psychosocial Care Center for four years or more, and 44.4% chose the mental health area. Analysis revealed three thematic axes: “Mental health in the academic context”; “Continuing education in mental health”; “Nursing role deconstruction or construction”.

**Conclusions:**

it was found that academic training is still insufficient to prepare nurses to work in the biopsychosocial model and expanded clinical practice, generating a feeling of unpreparedness and insecurity. In this scenario, continuing education emerges as an alternative to strengthen professional practice.

## RESUMEN

**Objetivos:**

comprender la percepción de enfermeras de la Secretaría de Salud del Distrito Federal y de residentes del Programa Multidisciplinario en Salud Mental Infantil y Adolescente sobre las prácticas del Centro de Atención Psicosocial Infantil y Adolescente, identificando desafíos y deficiencias en su formación.

**Métodos:**

se realizaron entrevistas semiestructuradas, y se analizaron mediante análisis temático, el cual consta de tres fases: preanálisis, exploración de contenido y elaboración de resultados.

**Resultados:**

de los participantes, el 55,5% ha trabajado en el Centro de Atención Psicosocial Infantil y Adolescente durante cuatro años o más, y el 44,4% eligió el área de salud mental. El análisis reveló tres ejes temáticos: “Salud mental en el contexto académico”; “Formación continua en salud mental”; “Deconstrucción o construcción del rol de enfermería”.

**Conclusiones:**

se constató que la formación académica aún resulta insuficiente para preparar enfermeras para trabajar en el modelo biopsicosocial y en la práctica clínica ampliada, lo que genera una sensación de falta de preparación e inseguridad. En este contexto, la formación continua se presenta como una alternativa para fortalecer la práctica profesional.

## INTRODUCTION

Historically, the concept of madness has had many interpretations, with the hygienist view being the most common from the 19^th^ century onwards. During this period, madness was seen as indiscipline, and people considered insane were isolated to maintain social peace. Therefore, asylums emerged as places of treatment. In the 1970s, the Psychiatric Reform movement, inspired by Franco Basaglia, began to question diagnoses and institutionalizations, which instigated demedicalization, self-care, and stigma reduction, marking the medical-hospital-centric-medicine model deinstitutionalization^(1-3)^.

Since then, there have been several advances driven by the reform movement, and in 2001, Federal Law 10,216 was enacted, proposing a new form of mental healthcare that replaces the hospital-centric model^(4)^. In this context, the Psychosocial Care Centers (In Portuguese, *Centros de Atenção Psicossocial* - CAPSs) emerged as an essential tool for the Brazilian Psychiatric Reform, seeking to restore citizenship and human dignity by producing new models of intervention^(5)^.

The CAPS, regulated by Ministerial Decree 336/2002, is a service that acts as a substitute for psychiatric hospitals, serving people with severe and persistent mental disorders. Specifically, the Child and Adolescent Psychosocial Care Center (In Portuguese, *Centro de Atendimento Psicossocial Infantojuvenil* - CAPSi), aimed at children and adolescents aged 0 to 18 years with severe psychological distress, offers individual, family, and group care, as well as therapeutic activities, visits, and coordination with other services, including referrals to primary and tertiary care. The CAPSi multidisciplinary team consists of at least 11 professionals, including physicians, nurses, psychologists, occupational therapists, and technicians, who work in an interdisciplinary manner^(6-9)^.

According to Pereira *et al*.^(10)^, nursing is recognized as an important workforce to meet the diverse demands of the Brazilian Health System (In Portuguese, *Sistema Único de Saúde* - SUS), motivating educational institutions to advocate for generalist training, capable of understanding and acting on the various dimensions and complexities of human beings. In this mental health context, nursing plays an essential role in the multidisciplinary team’s routine. However, there are many challenges when considering the history of hospital-centric and asylum-based practices in which nursing was inserted, under the hegemony of medical knowledge, with interventions that restricted patients’ behavior and individual characteristics^(11,12)^.

Especially in the field of child and adolescent mental health, there are even more complex specificities that require systemic care within the family and the care network in which children or adolescents are involved, in addition to the growth and development issues already studied in pediatric care courses. The regulation of CAPSis in Brazil is even more recent^(6)^. The perspective of children and adolescents experiencing psychological distress as bearers of rights and desires, and therefore involved in their demand for mental healthcare, proposes a change in relation to the practices that constituted the previously predominant model in the health field.

With the shift to the psychosocial model, nurses’ work requires readaptation and re-signification of their knowledge and practices, developing new scenarios and paths, changing the clinical perspective to a human perspective. Old therapeutic interventions have been updated through dialogue and listening, which have been important in the care provided by nursing for building bonds, affection, acceptance, and team intervention, caring not only for an individual, but for a family and a system^(13,14)^.

Knowing the need to develop new skills and competencies, and considering that the SUS has a great responsibility in the training of various healthcare professionals, CAPSs become settings for the practical training of undergraduate students and also for professionals in multidisciplinary residency programs at the graduate level (*lato sensu*), who seek to provide a training process based on welcoming, humanization, multi-professional teamwork, and user autonomy, in accordance with the SUS guiding principles. For residency programs in the mental health field, it is essential that residents work in CAPSs to understand the biopsychosocial model, learn about the functioning of the service, be prepared for various situations in public healthcare, and identify their strengths and weaknesses, contributing to the training of specialists^(15-17)^.

Given the above and the changes in the perspective of nursing care in mental health, this work seeks to explore the perspectives and difficulties encountered in nursing practice, from the perspective of public sector nursing professionals and resident nurses of the Multidisciplinary Residency Program in Child and Adolescent Mental Health, working in the CAPSis that comprise the Federal District’s Psychosocial Care Network (In Portuguese, *Rede de Atenção Psicossocial* - RAPS).

## OBJECTIVES

To understand the current perspectives of public sector nursing professionals and resident healthcare professionals (RHPs) working within the Child and Adolescent RAPS, especially in CAPSi, investigating possible difficulties related to the training model and practical application in the expanded clinical approach to the care of children and adolescents experiencing psychological distress.

## METHODS

### Ethical aspects

This research, originating from a Final Course Project of a residency program, meets the requirements set forth in the Guidelines and Regulatory Standards for Research Involving Humans^(18)^. It was approved by the *Fundação de Ensino e Pesquisa em Ciências da Saúde* Research Ethics Committee. After acceptance, all participants signed the Informed Consent Form in duplicate.

### Study design

This is a qualitative study, as it investigates beliefs, principles, and opinions on the subject matter, valuing the language and practice of the individuals involved^(19)^. To ensure quality in the preparation of this work, the COnsolidated criteria for REporting Qualitative research guide was used, composed of three indicator domains: 1) Research team and reflexivity; 2) Study concept; and 3) Analysis and results^(20)^.

### Methodological procedures

The data were obtained through semi-structured interviews, which allow for a description of the topic studied and an understanding of the perspectives of those involved. These interviews follow a guide with main questions, and supplementary questions may be included as needed^(19,21)^.

The recordings were converted into text and examined according to the thematic analysis method proposed by Minayo^(19)^, going through three phases: pre-analysis (reading and identification of core meanings); content exploration (creation of categories); and elaboration of results (analysis and confirmation of data according to the study theory and objectives).

### Study setting

The research was conducted at four CAPSis located in the Federal District (Recanto das Emas, Taguatinga, Asa Norte, and Sobradinho), which are part of RAPS, according to the regulations of Ordinance 3,088 of 2011^(22)^. These services cater to young people and children experiencing psychological distress and/or needs arising from the use of alcohol and other drugs, and operate in accordance with SUS’s training proposal, relying on professionals from the Multidisciplinary Residency Program in Mental Health for Children and Adolescents.

### Data source - sample

This study includes nurses employed by the Federal District Health Department working at the four CAPSis and nursing RHPs from the Multidisciplinary Residency Program in Child and Adolescent Mental Health assigned to the CAPSi setting at the time of the research.

Professionals who were on vacation or medical leave at the time of data collection were excluded. Resident professionals who had not worked in a CAPSi up to the time of data collection did not participate in the research.

The sample consists of 18 professionals, including two nursing RHPs from the Multidisciplinary Residency Program in Child and Adolescent Mental Health and 16 public employees from the Federal District Health Department working in Federal District CAPSis, with six from CAPSi Taguatinga, two from CAPSi Asa Norte, three from CAPSi Sobradinho, and five from CAPSi Recanto das Emas. The initial sample size was defined by convenience selection, in which the researcher chooses available participants^(23)^. The final size was determined using saturation sampling, stopping new inclusions when the data became repetitive^(24)^.

### Data collection and organization

The interviews were semi-structured, with questions about participants’ work history: length of professional experience in mental health, in CAPSis or other institutions; current position in the service; whether they had completed any training courses in mental health; and an open-ended question for reflection on professionals’ perceptions of their career path in mental health and in CAPSis, difficulties faced, participants’ choice of work location within the Federal District Health Department, and their undergraduate background.

Conducted separately, the interviews took place in private spaces or locations preferred by the interviewees. Recordings were made using the mobile app “Easy Voice Recorder”, and the transcribed excerpts were organized in Google Sheets. The Informed Consent Form was signed in duplicate.

### Data analysis

At the end of the research, the thematic axes extracted from the investigation were analyzed, namely: “Mental health in the academic context”; “Continuing education in mental health”; and “Nursing role deconstruction or construction”.

## RESULTS

Most participants have worked at CAPSi for four years or more (55.5%), and only 44.4% of respondents reported that they chose to work in mental health, including resident professionals.

Subsequently, we will address the tensions and final thematic axes extracted from the investigation, entitled: “Mental health in the academic context”; “Continuing education in mental health”; and “Nursing role deconstruction or construction”.

### Mental health in the academic context

In the first theme, participants recount their experiences with mental health education during their nursing degree and the difficulties they initially faced.

*I took the nursing course from 1998 to 2001, so it was an experience exactly at a time when there weren’t even substitute services, so, in my training, I also didn’t have any training geared towards that type of service.* (P13)

*It was very difficult, you know, because of the level of subjectivity, because the nursing degree is very objective, it’s very full of protocols, of guidelines, and so I felt a little lost, because there wasn’t, you know, in mental health, there isn’t a SOP* [Standard Operating Procedure]. *You don’t have a direction, you go by subjectivity, then you study, you go by experience.* (P16)

*And the nurse, from the beginning, from the first day of class to the last, we are studying physical* [...] *in this case, if a person really likes mental health, they have to do specialization on top of specialization in that.* (P8)

The collected statements reveal, in general, a significant mismatch between academic training in nursing and mental healthcare demands, especially in the context of the practice fostered by the Psychiatric Reform.

### Continuing education in mental health

In this second axis, we consider the relevance of continuing education, recognized by participants, regarding the percentage of respondents who did not choose to work in mental health (56.6%) and the impacts of this choice, as commented on by participants:

*So, I landed there by chance, right? But I took a deep breath when I saw that I was appointed to a CAPS, you know? I took a deep breath* [...]. *(P2)*

[...] *all the employees who were called at that time went to CAPS. You didn’t have much choice. At first, I even tried to leave, I couldn’t* [...] *at first, I was really scared, I didn’t really know what to do.* (P6)

*When we take on civil service exams, the courses we take, the training, some things like that aren’t taken into consideration much* [...]. (P5)

*I really miss continuing education. That’s what I see as the difference. At the hospital, we always had continuing education.* (P9)

*Sometimes, we can’t reconcile the training day with the schedule, availability, and so on. But there were some that were offered* [...] *that management is always concerned about being in this education process, right?* (P14)

We have noticed a recurring criticism regarding the lack of preparation and institutional support, highlighting the deficiency in continuing education. In addition to this, another topic to be discussed is the recognition by professionals of the need for continuing education in relation to their chosen field of work.

*Normally, it’s meetings, lectures, you know* [...] *occasional events, but only on my part. I’m not interested in taking courses, graduate studies, or more in-depth courses in the area of mental health, because it wasn’t a field I chose.* (P10)

### Nursing role deconstruction or construction

In this area, the interviews identified a common aspect among most participants regarding their role as nurses at CAPSi. They reported having gone through a journey of deconstructing the biomedical, hospital-centric, curative role of nursing in order to construct a biopsychosocial role, with a broader clinical approach and centered on the person being assisted.

*Very difficult. No, no. To this day, I still have difficulty understanding what the nurse does here. Although, well, I understand that everyone is a* [...]. *I say that here we are all therapists, right? But it’s difficult, isn’t it* [...]. (P11)

*I ask myself all the time. What am I going to do here? What do I have to do here? Because what I’m prepared as a nurse to do. It doesn’t seem to fit here at CAPSI. It seems! Because, little by little, you discover that this care goes much further, that it’s the care of the body as a whole, of self-care, right* [...] *of the interpersonal relationships that you can be working on with this patient, which I’ve been discovering* [...]. (P4)

Given this, both staff and residents similarly expressed discomfort regarding the deconstruction of nursing practice and care, especially when compared to hospital or outpatient settings.

*We move away a little from the actual nursing care because we are very focused on the biomedical field, on addressing physical problems, clinical problems. And here we move away from that a lot, right?* [...] *everyone becomes a therapist. I think that’s how it is here.* (P1)

*Because our role, we learn that it is very defined, right? We have a lot of protocols and everything. And here* [at CAPSi], *sometimes we don’t work with that, you know* [...] *so well defined for nursing.* (P7)

*Well, I think that, for every nurse, it’s very difficult to start working in an exclusive mental health environment, that only works with that, because we enter an identity crisis, right?* [...] *entering a scenario like this, without protocols, without anything very rigid, that ties you down, tightens you, puts you in a position of control over what will happen* [...] *it’s very difficult.* (P18)

Beyond the individual aspects of the category’s performance, it is observed that experiencing interprofessionalism in this context is a rich experience, but at the same time disconcerting for professionals who have not obtained theoretical and experiential training in expanded multidisciplinary clinical care, as commented by some participants:

*In my head, it was very much a “box”. Like, “oh, the speech therapist is important, the psychologist is important, and the nurse* [...] *and the nurse?”* [...] *so, this “box” view bothered me* [...]. (P3)

[...] *at first, I didn’t even know what CAPSI was. I didn’t know exactly what the different things were, what the care would actually be like, a nursing consultation within CAPS, so I still had a lot of this view of the boxes of specialties* [...]. (P13)

In this context, seeking a particular and characteristic performance of nursing, there were some reflections on how to apply the Nursing Process (NP) in children’s and adolescents’ mental health. It is possible to see that most of the professionals interviewed presented some doubts about how to apply the specific nursing tools developed to systematize, unify and support the care developed in the entire socio-environmental context, implemented and regulated by Resolution of the Federal Nursing Council 736 of January 2024^(25)^ on the NP.

*Now, how do you translate the Nursing Process, right? The data collection, the diagnosis, the planning, the implementation, the evaluation within the context of mental health, right? And how do you evolve that, right? So, I think it was really a learning experience, observing the daily routine of the services* [...]. (P15)

*I didn’t see, in practice, at first, any nurse who actually used SAE.* [...] *but that’s it, you know* [...] *I went into it by doing it and already incorporating this multidisciplinary aspect. And then, within all that we’ve been talking about, you know* [...] *the SAE, the Nursing Process* [...] *I ended up not putting it into practice. In my daily work, I’m more focused on the psychosocial approach.* (P17)

*It’s possible to do the Nursing Process, right? You can do anamnesis. You can make a nursing diagnosis. Establish treatment plans. And some of these plans are individual, which are your guidelines, and others are very collective and interdisciplinary, right, which is the group, which is how it will go through here. The PTS together with patients. That’s it.* (P12)

## DISCUSSION

Based on the results presented in the axes, we observed that, despite the topic being addressed, the training of interviewees does not correspond to the current demand for child and adolescent mental healthcare. This may be linked to the fact that some of the nurses completed their higher education before the creation of RAPS and alternative services. The complexity, uniqueness, and specificity of mental healthcare could not be fully captured in the clinical training characteristic of undergraduate courses in the profession.

Ito *et al*.^(26)^ argue that changes in nursing education are influenced by the needs of the time, often geared towards the hospital model of curative and individual care. This paradigm was present during the Psychiatric Reform, when therapy was controlled by medical-psychiatric discourse, aiming at social control and removing individuals’ autonomy^(27)^.

The hospital-centric, impersonal, and decontextualized characteristics of the comprehensive and holistic logic of mental healthcare taught in universities are reinforced by Tavares, who recognizes the essential role of nursing in improving the care provided in these services. To this end, she affirms the need to incorporate training and continuing education into the profession’s work process, with knowledge sharing throughout RAPS, in order to consolidate the Psychiatric Reform^(28)^.

With the establishment of the SUS and its guiding principles of equity, comprehensiveness, and universality, the Brazilian National Curriculum Guidelines for undergraduate health courses now have the new objective of training professionals in this new model. The higher education institutions’ responsibility in training professionals who can act and support the process of change in modern nursing, adapting it to the Brazilian society’s needs and the job market, has become clear^(26)^.

However, Reinaldo, Sousa, and Silveira identified weaknesses that still exist in the curriculum of nursing undergraduate programs, which offer mental health subjects with reduced hours and that do not correspond to the reality of care provided by RAPS. Furthermore, they pointed out that the faculty lack training in the area, which contributes to inconsistent teaching and the perpetuation of asylum-based, welfare-oriented, and hospital-centric practices^(29)^. It is important to emphasize that the current curriculum guidelines for undergraduate nursing programs date from 2001, and the ordinance establishing RAPS (Ordinance 3,088) is from 2011, a fact that may contribute to outdated teaching in this undergraduate program.

Therefore, academic training must prepare nursing professionals for the psychosocial model, which goes beyond traditional resources and practices, entering into an expanded clinical approach where the focus shifts to individuals, while the illness is placed in parentheses, allowing the perspective to move beyond being exclusively technical and clinical. Thus, an essential practice in the field of mental health is to center on individuals experiencing psychological distress-an individual who is biological, social, and subjective-instead of focusing solely on the illness^(30)^.

This complex form of practice can involve many personal issues for nurses. Zgiet reflects on the repercussions of professional well-being when it comes to working in mental health, which is not just a subsector and area of practice in health, but a field viewed under various stigmas that affect both patients and professionals. This is a circumstance capable of generating anxieties due to the various difficulties encountered, both in relation to institutional organization and in relation to the lack of resources and the feeling of professional inadequacy^(31)^.

In this regard, the changes in mental healthcare were encouraged by the unhealthy and uncomfortable conditions felt by patients and healthcare professionals, both leading actors of the Anti-Asylum Movement^(31)^. Therefore, the importance of an affinity with mental health and the desire to work in the field are highlighted so that professionals continue to be persistent advocates for humanized services, through an ethical, political, and affective commitment.

Therefore, in order to integrate professionals with the Psychiatric Reform practices and ideals, one of the components of the guidelines for the functioning of RAPS, as foreseen in Ordinance 3,088/2011, is continuing education^(22)^. According to interviewees’ reports, it is understood that only in some services does direct management actively offer this support to the team, in contrast to other reports that demonstrate a significant lack of this support. Furthermore, there were difficulties in reconciling the schedules of caregiving work and participation in these situations.

According to Tavares, education is also a process that acts on personal development to integrate and participate in society, and to be established, concrete needs must be analyzed. In this way, workers who recognize this need and are involved in the society in which they live seek to improve the quality of their work, working while simultaneously educating themselves^(28)^.

Therefore, continuing education in health presents itself as an important resource to foster changes in the mental health work process proposed by the Psychiatric Reform, since this is a living, complex, and transformative trajectory of relationships. In this process, the participation of professionals and individuals with knowledge about psychological distress and its social context is necessary, aiming at the construction of public policies that reinforce the sustainability of this new model of care^(32)^.

On the other hand, it is understood that changes in mental health practice may face resistance from professionals, as they encourage them to look critically at rigid and habitual routines in order to problematize them. This discomfort, when addressed within the team, allows professionals to look at themselves and allow themselves to learn again, creating new ways of producing knowledge and care focused on the needs of the individuals targeted by healthcare^(33)^.

Seeking to clarify and outline the challenges that nursing faces in mental health practice, Santos *et al*. affirm the complexity of this care when considering the singularities and peculiarities of each young person living with psychological distress. Similarly, care also involves change, going beyond procedures and practices associated solely with medication administration, physical restraint, hygiene, and feeding, to include multidisciplinary therapeutic activities and social reintegration^(12)^.

Referring to the last thematic axis (nursing role deconstruction or construction), it is a fact that multidisciplinary teams are advocated by policies and established in Healthcare Networks as an important component in the Psychiatric Reform consolidation. However, some perceptions continue to emphasize the individual practices of specialties and the biomedical model. Pinheiro *et al*. point out that the perpetuation of this conduct hinders the joint production of knowledge and practices in interdisciplinary work. Alternatively, a multidisciplinary team working together collaboratively creates space for discussions of anxieties and concerns that arise during the work process, enabling them to address the challenges of healthcare production^(34)^.

However, to have a unique role even as part of a multidisciplinary effort, Monteiro *et al*. write about the importance of applying a category-specific instrument in mental healthcare. This implies recognizing the profession as a science of care, as well as promoting improved care through the scientific and theoretical foundations of nursing. Such an instrument allows professionals to have clarity about their role in a grounded and unified manner, through the NP stages, promoting success in achieving the objectives. The authors also emphasize the importance of nursing being open to the diverse possibilities of care that exist in mental health, learning to engage critically in the dialogue about the Psychiatric Reform and madness, about the objective and the subjective, and being available to deconstruct representations of psychological distress^(35)^.

### Study limitations

This study presents some limitations that should be considered when interpreting the results. Among them, the small number of nursing RHPs may have impacted the scope of the analyses, reducing the diversity of perspectives and experiences collected. In this regard, studies capable of including a more representative overview of the class’s perception would allow for a more elaborate analysis of the findings.

Similarly, the scarcity of previous studies on the subject made it difficult to contextualize the theoretical and comparative aspects of results. The lack of consolidated references demanded an extra effort in developing the theoretical framework and supporting the analyses, which may have restricted the complexity of the interpretations.

These limitations do not invalidate the research results, but they signal the importance of future studies that increase the number of participants and deepen the investigation into the subject, helping to provide a more complete and well-founded understanding.

### Contributions to nursing and mental health

This study makes relevant contributions to nursing by highlighting the need for updates in the professional training and clinical practice of nurses working in child and adolescent mental health, aligning theory and practice. Furthermore, it emphasizes the need for continuing education as an essential tool for professional development, providing greater security and preparedness to deal with the complexities of the field.

For the population, the qualification of professionals directly reflects on quality of care, making it more humanized, safe, and patient-centered, guaranteeing assistance in accordance with the RAPS demands and guidelines.

## FINAL CONSIDERATIONS

The results highlighted the dichotomy between the knowledge acquired in undergraduate training and the clinical practice required by the current policy and organization of mental healthcare. The magnitude of the role of educational institutions as trainers of professionals and promoters of critical, curious, and reflective knowledge cannot be overlooked. There is a need for improvements and updates in the teaching process to guarantee the necessary knowledge to work with the complexities of the individuals assisted in mental health units.

This lack of preparation in professional training, combined with personal choices about the area of practice, can lead to what has been discussed, such as the deconstruction or construction of the role and work process of nursing, in which professionals undergo a reform of clinical care practice, experiencing insecurities, questioning, and professional depersonalization.

Among the resources available to support this need are continuing education and continuing training, in a manner consistent with the reality of nurses and professional demands, to operate within the logic of RAPS. However, the difficulties encountered in implementing continuing education are understandable, whether due to adversities in the organization of its application or the discomfort resulting from critically examining one’s own work process. Furthermore, the effective use of a work process that systematizes and unifies care in a way that adapts to individuals’ needs, such as NP, can help safeguard professional practice, grounded in both theory and practice.

## Data Availability

The research data are available only upon request.
