# Supplementary material for: Perspectives on the care process by nurses working in Child and Adolescent Psychosocial Care Centers
Source: Rev Bras Enferm. 2026 Jul 6;79:e20250156. doi: 10.1590/0034-7167-2025-0156 (PMC13336225; doi:10.1590/0034-7167-2025-0156)
Supplement: Translation

# Translation: Perspectivas sobre o processo de cuidado pela enfermagem atuante nos Centros de Atenção Psicossocial Infantojuvenil

**DOI:** 10.1590/0034-7167-2025-0156pt

**Authors:** Fernanda da Costa Ferreira, Mariana Miguel Vieira, Ângela Maria Rosas Cardoso

**Affiliations:** I Fundação de Ensino e Pesquisa em Ciências de Saúde. Brasília, Distrito Federal, Brasil

**Keywords:** Serviços de Saúde Mental, Enfermagem, Enfermagem Psiquiátrica, Modelos Biopsicossociais, Assistência à, Saúde Mental.

## Abstract

**Objetivos:**

compreender a percepção de enfermeiros da Secretaria da Saúde do Distrito Federal e residentes do Programa Multiprofissional de Saúde Mental Infantojuvenil sobre práticas no Centro de Atenção Psicossocial Infantojuvenil, identificando desafios e lacunas na formação.

**Métodos:**

foram realizadas entrevistas semiestruturadas, analisadas segundo a técnica de análise temática, composta por três fases: pré-análise, exploração do conteúdo e elaboração dos resultados.

**Resultados:**

dos participantes, 55,5% atuam no Centro de Atenção Psicossocial Infantojuvenil há quatro anos ou mais, e 44,4% escolheram a área da saúde mental. A análise revelou três eixos temáticos: “A saúde mental no contexto acadêmico”; “A educação permanente na saúde mental”; “A desconstrução ou construção do papel da enfermagem”.

**Conclusões:**

constatou-se que a formação acadêmica ainda é insuficiente para preparar os enfermeiros para atuar no modelo biopsicossocial e clínica ampliada, gerando sensação de despreparo e insegurança. Nesse cenário, a educação permanente surge como alternativa para fortalecer a prática profissional.

## INTRODUÇÃO

Historicamente, o conceito de loucura teve muitas interpretações, sendo a visão higienista a mais comum a partir do século XIX. Nesse período, a loucura era vista como indisciplina, e as pessoas consideradas loucas eram isoladas para manter a paz social. Portanto, surgiram os hospícios como locais de tratamento. Na década de 70, o movimento da Reforma Psiquiátrica, inspirado por Franco Basaglia, começou a questionar diagnósticos e institucionalizações, o que instigou a desmedicalização, autocuidado e redução do estigma, marcando a desinstitucionalização do modelo médico-hospitalocêntrico-medicamentoso^(1-3)^.

Desde então, houve vários avanços impulsionados pelo movimento da reforma, e em 2001, foi sancionada a Lei Federal n^o^ 10.216, que propõe uma nova forma de assistência à saúde mental substitutiva ao padrão hospitalocêntrico^(4)^. Nesse contexto, surgem os Centros de Atenção Psicossocial (CAPSs), um dispositivo essencial para a Reforma Psiquiátrica Brasileira, que buscam resgatar a cidadania e dignidade humana, produzindo novos modelos de intervenção^(5)^.

O CAPS, regulamentado pela Portaria do Gabinete Ministerial nº 336/2002, é um serviço que atua de maneira substitutiva aos manicômios, atendendo pessoas com transtornos mentais severos e persistentes. Em específico, o Centro de Atendimento Psicossocial Infantojuvenil (CAPSi), voltado a crianças e adolescentes de 0 a 18 anos com sofrimento psíquico grave, oferece atendimentos individuais, familiares e em grupo, além de atividades terapêuticas, visitas e articulação com outros serviços, incluindo matriciamentos com atenção primária e terciária. A equipe multiprofissional do CAPSi é composta por, no mínimo, 11 profissionais, incluindo médicos, enfermeiros, psicólogos, terapeutas ocupacionais e técnicos, que atuam de forma interdisciplinar^(6-9)^.

Conforme Pereira *et al*.^(10)^, a enfermagem é reconhecida como uma importante força de trabalho para atender demandas diversas do Sistema Único de Saúde (SUS), motivando as instituições de ensino a preconizar a formação generalista, capaz de entender e atuar nas várias dimensões e complexidades do ser humano. Nesse contexto de saúde mental, a enfermagem tem um papel essencial na rotina da equipe multiprofissional. Entretanto, há muitos desafios ao considerarmos o histórico das práticas hospitalocêntricas e manicomiais em que a enfermagem foi inserida, sob a hegemonia do saber médico, com intervenções que restringiam o comportamento e singularidades dos pacientes^(11,12)^.

Especialmente no campo da saúde mental infantil e adolescente, existem especificidades ainda mais complexas que exigem cuidados sistêmicos no âmbito familiar e na rede de cuidados em que a criança ou o adolescente está inserido, além das questões de crescimento e desenvolvimento já estudadas nas matérias de cuidados pediátricos. A regulamentação dos CAPSis é no Brasil ainda mais recente^(6)^. A perspectiva de crianças e adolescentes em sofrimento psíquico como portadores de direitos e desejos, e, portanto, envolvidos em sua demanda por cuidados de saúde mental, propõe uma mudança em relação às práticas que constituíam o modelo anteriormente predominante na área da saúde.

Com a passagem para o modelo psicossocial, o trabalho do enfermeiro necessita de readaptação e ressignificação dos seus saberes e práticas, desenvolvendo novos cenários e caminhos, mudando o olhar clínico para um olhar humano. Atualizaram-se as antigas intervenções terapêuticas através do diálogo e da escuta, os quais têm sido importantes no cuidado proporcionado pela enfermagem para construção do vínculo, do afeto, do acolhimento e da intervenção em equipe, cuidando não só de uma pessoa, mas de uma família e de um sistema^(13,14)^.

Sabendo sobre a necessidade de desenvolver novas habilidades e competências, e considerando que o SUS tem grande responsabilidade na formação para diversos profissionais de saúde, os CAPSs se tornam cenários para práticas de discentes de cursos de graduação e também para profissionais dos programas de residências multiprofissionais ao nível de pós-graduação (*lato sensu*), que buscam proporcionar um processo formativo fundamentado no acolhimento, humanização, ação em equipe multiprofissional e autonomia do usuário, conforme os princípios norteadores do SUS. Para os programas de residência na área da saúde mental, é essencial que os residentes atuem em CAPSs para compreender o modelo biopsicossocial, conhecer o funcionamento do serviço, ser preparado para diversas situações na assistência na saúde pública, e identificar suas potencialidades e fragilidades, contribuindo para a formação de especialistas^(15-17)^.

Diante do exposto e das mudanças na perspectiva dos cuidados de enfermagem em saúde mental, o trabalho em questão busca explorar as perspectivas e dificuldades encontradas na atuação da enfermagem, na perspectiva dos enfermeiros servidores e dos enfermeiros residentes do Programa Multiprofissional de Residência em Saúde Mental Infantojuvenil, atuantes nos CAPSis que compõem a Rede de Atenção Psicossocial (RAPS) do Distrito Federal.

## OBJETIVOS

Compreender as perspectivas atuais dos profissionais de enfermagem funcionários públicos e profissionais de saúde residentes (PSRs) inseridos na RAPS infantojuvenil, especialmente no CAPSi, investigando as possíveis dificuldades em relação ao modelo de formação e à atuação prática na proposta de clínica ampliada para os cuidados à criança e adolescente em sofrimento psíquico.

## MÉTODOS

### Aspectos éticos

A presente pesquisa, oriunda de Trabalho de Conclusão de Curso de programa de residência, atende aos requisitos dispostos nas Diretrizes e Normas Regulamentadoras das Pesquisas Envolvendo Humanos^(18)^. Foi aprovada pelo Comitê de Ética em Pesquisa da Fundação de Ensino e Pesquisa em Ciências da Saúde. Após o aceite, todos os participantes assinaram o Termo de Consentimento Livre e Esclarecido em duas vias.

### Tipo de estudo

Trata-se de estudo qualitativo, visto que investiga crenças, princípios e opiniões sobre o tema em questão, valorizando a linguagem e prática dos indivíduos envolvidos^(19)^. Para garantir a qualidade na elaboração deste trabalho, utilizou-se o guia *COnsolidated criteria for REporting Qualitative research*, composto por três domínios de indicadores: 1) Equipe de pesquisa e reflexividade; 2) Conceito do estudo; e 3) Análise e resultados^(20)^.

### Procedimentos metodológicos

Os dados foram obtidos por meio de entrevistas semiestruturadas, que possibilitam descrever o tema estudado e a compreender a parte dos envolvidos. Essas entrevistas seguem um guia com questões principais, podendo incluir perguntas complementares conforme a necessidade^(19,21)^.

As gravações foram convertidas em texto e examinadas conforme o método de análise temática proposta por Minayo^(19)^, passando por três fases: pré-análise (leitura e identificação de núcleos de sentido); exploração de conteúdo (criação de categorias); e elaboração dos resultados (análise e confirmação dos dados de acordo com a teoria e os objetivos do estudo).

### Cenário do estudo

A pesquisa foi conduzida em quatro CAPSis localizados no Distrito Federal (Recanto das Emas, Taguatinga, Asa Norte e Sobradinho), que fazem parte da RAPS, conforme a regulamentação da Portaria nº 3.088 de 2011^(22)^. Esses serviços atendem jovens e crianças em sofrimento mental e/ou necessidades decorrentes do uso de álcool e outras drogas, e funcionam em consonância com a proposta formativa do SUS, contando com profissionais do Programa de Residência Multiprofissional em Saúde Mental para Infância e Adolescência.

### Fonte de dados - amostra

Participam deste estudo os enfermeiros servidores da Secretaria da Saúde do Distrito Federal atuantes nos quatro CAPSis e os PSRs de enfermagem do Programa de Residência Multiprofissional em Saúde Mental Infantojuvenil lotados no cenário de CAPSi no momento da pesquisa.

Foram excluídos os profissionais que estavam de férias ou de licença médica no momento da coleta de dados. Os profissionais residentes que não tenham atuado em um CAPSi até o momento da coleta de dados não participaram da pesquisa.

A amostra é composta por 18 profissionais, sendo dois PSRs de enfermagem do Programa de Residência Multiprofissional em Saúde Mental Infantojuvenil e 16 funcionários públicos da Secretaria da Saúde do Distrito Federal lotados nos CAPSis do Distrito Federal, sendo seis do CAPSi Taguatinga, dois do CAPSi Asa Norte, três do CAPSi Sobradinho e cinco do CAPSi Recanto das Emas. O tamanho inicial da amostra foi definido por seleção por conveniência, em que o pesquisador escolhe participantes disponíveis^(23)^. Já o tamanho final seguiu a amostragem por saturação, interrompendo novas inclusões quando os dados se tornaram repetitivos^(24)^.

### Coleta e organização dos dados

As entrevistas ocorreram de modo semiestruturado, com perguntas sobre os dados laborais do participante: tempo de atuação profissional na saúde mental, em CAPSis ou em outras instituições; cargo atual no serviço; se houve a realização de cursos capacitantes realizados na área da saúde mental; e uma indagação para reflexão aberta acerca das percepções do profissional sobre a sua trajetória na saúde mental e no CAPSi, dificuldades enfrentadas, a escolha do participante pelo local de atuação na Secretaria da Saúde Distrito Federal e o seu histórico na graduação.

Conduzidas separadamente, as entrevistas aconteceram em espaços privados ou em locais preferidos pelos entrevistados. As gravações foram realizadas através do um aplicativo de celular Gravador de Voz Fácil, e os trechos transcritos foram organizados em Planilhas Google. O Termo de Consentimento Livre e Esclarecido foi assinado em duas cópias.

### Análise dos dados

Ao final da pesquisa, foram analisados os eixos temáticos extraídos da investigação denominados: “A saúde mental no contexto acadêmico”; “A educação permanente na saúde mental”; e “A desconstrução ou construção do papel da enfermagem”.

## RESULTADOS

A maioria dos participantes trabalham no CAPSi há quatro anos ou mais (55,5%), e apenas 44,4% dos entrevistados relataram que escolheram trabalhar na saúde mental, incluindo os profissionais residentes.

A seguir, trataremos sobre as tensões e os eixos temáticos finais extraídos da investigação denominados: “A saúde mental no contexto acadêmico“; “A educação permanente na saúde mental”; e “A desconstrução ou construção do papel da enfermagem”.

### A saúde mental no contexto acadêmico

No primeiro tema, os participantes narram suas experiências sobre a educação em saúde mental durante a graduação de enfermagem e as dificuldades inicialmente enfrentadas.

*Eu fiz o curso de enfermagem de 1998 a 2001, então foi uma vivência exatamente numa época que nem se tinha serviços substitutivos, então, na minha formação, eu também não tive nenhuma capacitação voltada para esse tipo de serviço.* (P13)

*Eu, assim, foi muito difícil, né, pelo nível da subjetividade, porque a graduação da enfermagem, ela é muito objetiva, ela é muito cheia de protocolos, de diretrizes, e aí eu me senti um pouco perdida, porque não tinha, né, na saúde mental, assim* [...] *não tem um POP* [Procedimento Operacional Padrão]. *Você não tem um norte, você vai pela subjetividade, aí você vai estudar, vai pela experiência.* (P16)

*E o enfermeiro, ele desde o início, desde o primeiro dia de aula até o último, a gente tá estudando físico* [...] *no caso, se a pessoa realmente gosta de saúde mental, ela tem que fazer especialização em cima de especialização naquilo.* (P8)

As falas coletadas revelam, no geral, um descompasso significativo entre a formação acadêmica em enfermagem e as exigências do cuidado em saúde mental, especialmente no contexto da prática fomentado Reforma Psiquiátrica.

### A educação permanente na saúde mental

Nesse segundo eixo, contemplamos a relevância da educação permanente, reconhecida pelos participantes, sobre a porcentagem de entrevistados que não escolheram atuar na saúde mental (56,6%) e os impactos dessa escolha, como comentado pelos participantes:

*Então, eu caí de paraquedas, né? Mas eu respirei fundo quando eu vi que eu fui nomeada pra um CAPS, sabia? Respirei fundo* [...]. (P2)

[...] *todos os servidores que foram chamados naquela época foram pra CAPS. A pessoa não podia muito escolher. No começo, eu até tentei sair, não consegui* [...] *no começo, realmente, fiquei com medo, não sabia muito bem o que fazer.* (P6)

*Quando a gente assume concursos, não é levado muito em consideração os cursos que a gente faz, as capacitações, algumas coisas assim* [...]. (P5)

*Eu sinto muita falta de uma educação continuada. O que eu vejo a diferença é isso. No hospital, a gente tinha sempre educação continuada.* (P9)

*Às vezes, a gente não consegue conciliar o dia da capacitação com o horário da escala, disponibilidade e tal. Mas teve algumas que foram oferecidas* [...] *que a gerência tem essa preocupação de sempre estar nesse processo de educação, né?* (P14)

Percebermos uma crítica recorrente sobre a ausência de preparo e apoio institucional, destacando a carência de educação continuada. Suplementar a isso, outro tópico a ser discutido é o reconhecimento da necessidade de educação permanente pelos profissionais associada à escolha da área de atuação.

*Normalmente, são reuniões, palestras, é* [...] *pontuais, mas da minha parte mesmo. Eu não tenho interesse em fazer cursos, pós-graduação, cursos mais aprofundados na área de saúde mental, porque não foi uma área que eu escolhi.* (P10)

### A desconstrução ou construção do papel da enfermagem

Nesse eixo, foi identificado nas entrevistas um aspecto em comum entre a maioria dos participantes sobre o seu papel como enfermeiro no CAPSi. Relatam ter passado por uma jornada de desconstrução do papel biomédico, hospitalocêntrico, curativo da enfermagem para construção do papel biopsicossocial, de clínica ampliada e centrado na pessoa que está sendo assistida.

*Muito difícil. Não, não. Até hoje, ainda tenho dificuldade de entender o que que o enfermeiro faz aqui. Apesar de, assim, eu entendo que todo mundo é um* [...]. *Eu falo que aqui todos nós somos terapeutas, né? Mas é difícil, né* [...]. (P11)

*Me pergunto a todo momento. O que que eu vou fazer aqui? O que que eu tenho para fazer aqui? Porque o que eu tô preparado como enfermeiro para fazer. Parece que não ajusta aqui no CAPSI. Parece! Porque, aos poucos, você vai descobrindo que esse cuidar vai muito mais além, que é o cuidado do corpo como um todo, do autocuidado, né* [...] *das relações interpessoais que você pode tá trabalhando com esse paciente, que eu venho descobrindo isso* [...] (P4)

Diante disso, tanto os profissionais servidores quanto residentes similarmente expressaram o desconforto experienciado sobre a desconstrução da atuação e do cuidado da enfermagem, especialmente quando comparados a instituições hospitalares ou ambulatoriais.

*A gente sai um pouquinho da assistência mesmo de enfermagem porque a gente é voltado muito para a atuação e para o biomédico, para atuação no problema físico, no problema clínico. E aqui a gente sai muito disso, né?* [...] *vira todo mundo terapeuta. Eu acho que aqui é assim.* (P1)

*Porque o nosso papel, a gente aprende que ele é muito definido, né? A gente tem muito protocolo e tudo. E aqui* [no CAPSi], *às vezes, a gente não trabalha com isso, né* [...] *assim, bem definido pra enfermagem.* (P7)

*Bom, eu acho que, para todo enfermeiro né, é bem difícil começar a trabalhar num ambiente de saúde mental exclusivo, que trabalhe só com isso, porque a gente entra numa crise de identidade, né?* [...] *entrar num cenário como esse, sem protocolos, sem nada assim muito rígido, que te amarra, te aperta, te coloca ali numa condição de controle do que vai acontecer* [...] *é bem difícil.* (P18)

Além dos aspectos individuais da atuação da categoria, observa-se que experienciar a interprofissionalidade nesse contexto é uma vivência rica, mas, ao mesmo tempo, desconcertante para profissionais que não obtiveram uma formação teórica e vivencial do cuidado de clínica ampliada multiprofissional, como comentado por alguns participantes:

*Na minha cabeça, era muito “caixinha”. Tipo, “ah, o fono tem importância, o psicólogo tem importância, e o enfermeiro* [...] *e o enfermeiro?”* [...] *então, essa visão de “caixinha” me incomodava* [...]. (P3)

[...] *num primeiro momento, também não sabia nem o que era CAPSI. Não sabia exatamente quais eram as coisas diferentes, o que seria o atendimento mesmo, uma consulta de enfermagem dentro do CAPS, então ainda tinha muito esse olhar das caixinhas das especialidades* [...]. (P13)

Nesse contexto, buscando uma atuação particular e característica da enfermagem, houve algumas reflexões sobre como aplicar o Processo de Enfermagem (PE) na saúde mental de crianças e adolescentes. É possível constatar que a maioria dos profissionais entrevistados apresentou certas dúvidas de como aplicar as ferramentas específicas da enfermagem desenvolvidas para sistematizar, unificar e apoiar o cuidado desenvolvido em todo o contexto socioambiental, implementado e regulado pela Resolução do Conselho Federal de Enfermagem nº 736 de janeiro de 2024^(25)^ sobre o PE.

*Agora, como fazer essa tradução, assim, do Processo de Enfermagem, né? A coleta de dados, o diagnóstico, o planejamento, a implementação, a avaliação dentro do contexto de saúde mental, né? E como evoluir isso, né? Então, acho que foi um aprendizado mesmo de ver a rotina do cotidiano dos serviços* [...]. (P15)

*Não vi na prática, né, assim de início, nenhum enfermeiro que utilizasse mesmo da SAE, né.* [...] *mas é isso, né* [...] *fui dentro de um fazer mesmo e já incorporando essa coisa do multiprofissional. E aí acabou que eu também, dentro disso tudo que a gente tem conversado, né* [...] *da SAE, do Processo de Enfermagem. Acabou que eu não vou colocando isso na prática. No meu dia a dia, eu vou mais dentro da lógica do psicossocial mesmo.* (P17)

*Dá pra fazer, né, o Processo de Enfermagem. Dá pra fazer anamnese. Dá pra fazer diagnóstico de enfermagem. Estabelecer condutas. E as condutas, algumas individuais, que são suas orientações, e outras bem coletivas e interdisciplinares, né, que é o grupo, que é como ele vai passar aqui dentro. O PTS junto com o paciente. É isso.* (P12)

## DISCUSSÃO

Observamos, pelos resultados apresentados nos eixos, que apesar de o assunto ter sido abordado, a formação dos entrevistados não condiz com a demanda atual do atendimento em saúde mental infantojuvenil. Isso pode estar ligado ao fato de que alguns dos enfermeiros finalizaram o ensino superior antes da criação da RAPS e dos serviços alternativos. A complexidade, a singularidade e a especificidade dos cuidados em saúde mental não puderam ser totalmente capturadas no treinamento clínico característico dos cursos de graduação da profissão.

Ito *et al*.^(26)^ afirmam que as mudanças no ensino de enfermagem são influenciadas pelas necessidades da época, frequentemente voltadas ao modelo hospitalar de atenção curativa e individual. Esse paradigma esteve presente durante a Reforma Psiquiátrica, quando a terapêutica era controlada pelo discurso médico-psiquiátrico, visando ao controle social e retirando a autonomia dos indivíduos^(27)^.

As características hospitalocêntricas, impessoais e descontextualizadas da lógica integral e holística da atenção à saúde mental ensinada nas universidades são reforçadas pela autora Tavares, que reconhece o papel essencial da enfermagem na melhoria da assistência prestada nesses serviços. Para tanto, ela afirma a necessidade de incorporar a formação e a educação continuada ao processo de trabalho da profissão, com compartilhamento de conhecimento em toda a RAPS, a fim de consolidar a Reforma Psiquiátrica^(28)^.

Com a instituição do SUS e dos princípios norteadores de equidade, integralidade e universalidade, as Diretrizes Curriculares Nacionais dos cursos de graduação em saúde passam a ter o novo objetivo de capacitar profissionais nesse novo modelo. Ficou clara a responsabilidade das instituições de ensino superior na formação de profissionais que possam atuar e amparar o processo de mudança na enfermagem moderna, adequando às necessidades da sociedade brasileira e do mercado de trabalho^(26)^.

Todavia, os autores Reinaldo, Sousa e Silveira identificaram fragilidades ainda existentes na grade curricular das graduações de enfermagem, que oferecem disciplinas de saúde mental com carga horária reduzida e que não correspondem à realidade da assistência pela RAPS, além de apontarem que os docentes não possuem formação na área, o que contribui para a inconsistência do ensino e para a perpetuação das práticas manicomiais, assistencialistas e hospitalocêntricas^(29)^. É importante ressaltar que as diretrizes curriculares vigentes para a graduação em enfermagem são de 2001, e a portaria que institui a RAPS (Portaria nº 3.088) é de 2011, um fato capaz de colaborar para desatualização no ensino desta graduação.

Portanto, a formação acadêmica deve preparar o profissional enfermeiro para o modelo psicossocial, que vai além dos recursos e práticas tradicionais, adentrando a clínica ampliada, em que o foco se desloca para o sujeito, enquanto a doença é colocada entre parênteses, permitindo que o olhar deixe de ser exclusivamente técnico e clínico. Assim, uma prática essencial no campo da saúde mental é se centrar no sujeito em sofrimento psíquico, um sujeito que é biológico, social e subjetivo, em vez de se concentrar apenas na doença^(30)^.

Essa forma complexa de atuação pode implicar muitas questões pessoais do enfermeiro. Zgiet reflete sobre as repercussões do bem-estar profissional, quando se trata de atuar na saúde mental, que não é apenas um subsetor e área de atuação na saúde, mas um campo visto sob vários estigmas que afetam os pacientes e os profissionais. Essa é uma circunstância capaz de gerar angústias devido às diversas dificuldades encontradas, tanto em relação à organização institucional quanto em relação à falta de recursos e ao sentimento de incapacidade profissional^(31)^.

Nesse sentido, as modificações no cuidado à saúde mental foram estimuladas pela insalubridade e desconforto sentidos pelos pacientes e profissionais de saúde, ambos protagonistas do Movimento da Luta Antimanicomial^(31)^. Por isso, destacam-se a importância da afinidade com a saúde mental e a importância do querer atuar na área, para que os profissionais continuem sendo defensores insistentes de serviços humanizados, por intermédio de um compromisso ético, político e afetivo.

Dessa forma, para integrar os profissionais com as práticas e ideais da Reforma Psiquiátrica, um dos componentes das diretrizes para o funcionamento da RAPS, previsto na Portaria nº 3.088/2011, é a educação permanente^(22)^. Conforme os relatos dos entrevistados, entende-se que apenas em alguns serviços a gestão direta oferece ativamente esse suporte para a equipe, em detrimento de outras falas, que demonstram uma carência importante desse suporte. Além disso, houve dificuldades de conciliar as escalas de trabalhos assistenciais e participação para esses momentos.

Conforme Tavares, a educação também é um processo que atua sobre o desenvolvimento pessoal para se integrar e ser participante na sociedade, e para ser estabelecido, devem-se analisar as necessidades concretas para tal. Dessa maneira, o trabalhador que reconhecesse essa necessidade e está envolvido na sociedade em que vive busca melhorar a qualidade de sua ocupação, trabalhando ao mesmo tempo em que se educa^(28)^.

Portanto, a educação permanente em saúde se apresenta como um recurso importante para fomentar mudanças no processo de trabalho em saúde mental proposta pela Reforma Psiquiátrica, já que esta é uma trajetória viva, complexa e transformadora de relações. Nesse percurso, é necessária a participação de profissionais e pessoas com conhecimentos sobre o sofrimento psíquico e seu lugar social, que visem à construção de políticas públicas que reforcem a sustentação desse novo modelo de cuidado^(32)^.

Por outro lado, entende-se que as mudanças na prática da saúde mental podem sofrer resistência por parte dos profissionais, pois os incentivam a olhar para as rotinas enrijecidas e habituais de maneira crítica para problematizá-la. Esse incômodo, ao ser trabalhado na equipe, permite que o profissional olhe para si e se permita ser aprendiz de novo para criar novas formas de produzir o saber e o cuidado focados nas necessidades do sujeito alvo da atenção à saúde^(33)^.

Buscando esclarecer e delinear esses desafios que a enfermagem enfrenta na prática em saúde mental, Santos *et al*. afirmam a complexidade dessa assistência ao olhar as singularidades e peculiaridades de cada jovem que convive com um sofrimento mental. Da mesma forma, o cuidado também sobre mudanças, ao ultrapassar procedimentos e práticas associadas somente à administração de medicamentos, contenção física, higiene e alimentação, incluindo-se atividades terapêuticas multidisciplinares e reinserção social^(12)^.

Fazendo inferência ao último eixo temático (desconstrução ou construção do papel da enfermagem), é fato que as equipes multiprofissionais são preconizadas pelas políticas e instituídas nas Redes de Atenção à Saúde como componente importante na consolidação da Reforma Psiquiátrica. Entretanto, algumas percepções continuam enfatizando as práticas individuais das especialidades e o modelo biomédico. Pinheiro *et al*. apontam que a perpetuação desta conduta dificulta a produção em conjunto de saberes e fazeres do trabalho interdisciplinar. De outro modo, uma equipe multiprofissional que atua de maneira conjunta e compartilhada abre espaço para discussões de angústias e inquietações que surgem durante o processo de trabalho, possibilitando enfrentamento das dificuldades da produção em saúde^(34)^.

Entretanto, para ter uma atuação privativa mesmo como parte de um trabalho multiprofissional, Monteiro *et al*. escrevem sobre a importância da aplicação de um instrumento próprio da categoria na assistência à saúde mental, o que implica o reconhecimento da profissão como uma ciência do cuidado, além de promover a melhoria da assistência através das bases científicas e teóricas da enfermagem. Um instrumento como esse possibilita que o profissional tenha clareza sobre sua atuação de maneira fundamentada e unificada, por meio das etapas do PE, promovendo êxito na realização dos objetivos. Os autores também enfatizam a importância de a enfermagem estar aberta às diversas possibilidades de cuidado que existem na saúde mental, aprendendo a se engajar criticamente no diálogo sobre a Reforma Psiquiátrica e a loucura, sobre o objetivo e o subjetivo, e estando disponível para desconstruir representações do sofrimento psíquico^(35)^.

### Limitações do estudo

Este estudo apresenta algumas limitações que devem ser ponderadas na interpretação dos resultados. Entre elas, o número reduzido de PSRs de enfermagem pode ter impactado a abrangência das análises, reduzindo a diversidade de perspectivas e experiências coletadas. Nessa direção, estudos com capacidade de incluir um panorama mais representativo da percepção da classe permitiriam uma análise mais elaborada dos achados.

Da mesma forma, a escassez de estudos prévios sobre a temática dificultou a contextualização teórica e comparativa dos resultados. A carência de referências consolidadas demandou um esforço extra na elaboração do arcabouço teórico e no suporte das análises, o que pode ter restringido a complexidade das interpretações.

Essas limitações não anulam os resultados da pesquisa, mas sinalizam a importância de estudos futuros que aumentem o número de participantes e aprofundem a investigação sobre o assunto, ajudando a proporcionar uma compreensão mais completa e bem fundamentada.

### Contribuições para as áreas da enfermagem e saúde mental

Este estudo traz contribuições relevantes para a enfermagem, ao evidenciar a necessidade de atualizações na formação profissional e na prática clínica dos enfermeiros que atuam na saúde mental infantojuvenil, com o alinhamento entre teoria e prática. Além disso, ressalta-se a necessidade da educação permanente como ferramenta essencial para qualificação profissional, proporcionando maior segurança e preparo para lidar com as complexidades da área.

Para a população, a qualificação dos profissionais reflete diretamente na qualidade do atendimento, tornando-o mais humanizado, seguro e centrado no paciente, garantindo uma assistência de acordo com as demandas e diretrizes da RAPS.

## CONSIDERAÇÕES FINAIS

Os resultados evidenciaram a dicotomia entre os saberes adquiridos na formação de graduação e a prática clínica exigida pela atual política e organização da assistência da saúde mental, não se pode passar despercebido a magnitude do papel das instituições de ensino enquanto formadoras de profissionais e promotoras de conhecimento crítico, curioso e reflexivo. Constata-se a necessidade de melhorias e atualizações no processo de ensino, para garantir o conhecimento necessário para atuar com as complexidades dos indivíduos atendidos nas unidades de saúde mental.

Esse despreparo na formação profissional, associado às escolhas pessoais sobre a área de atuação, pode ocasionar o que foi discutido, como a desconstrução ou a construção do papel e do processo de trabalho da enfermagem, no qual o profissional passa por uma reforma da prática clínica do cuidado, vivenciando inseguranças, questionamentos e despersonalização profissional.

Entre os recursos disponíveis para amparar essa necessidade, estão a educação continuada e a formação permanente, de maneira condizente com a realidade dos enfermeiros e das demandas profissionais, para atuar na lógica da RAPS. Mas compreendem-se as dificuldades encontradas na implementação da educação permanente, seja pelas adversidades na organização da aplicação ou pelo incômodo resultante de olhar para o próprio processo de trabalho de forma crítica. Em complemento, o uso efetivo de um processo de trabalho, que sistematiza e unifica o cuidado de maneira adaptável às necessidades do sujeito, como o PE, pode colaborar para resguardar a prática profissional, fundamentado de maneira teórica e prática.

## Data Availability

Os dados de pesquisa só estão disponíveis mediante solicitação.
